# Domain-specific associations of metabolomic features with cognition and neural networks among cognitively normal adults

**DOI:** 10.3389/fnins.2026.1898605

**Published:** 2026-10-20

**Authors:** Brandi L. Vollmer, Christian Habeck, Vrinda Kalia, Badri N. Vardarajan, Nikolaos Scarmeas, Gary W. Miller, Yaakov Stern, Yian Gu

**Affiliations:** 1 Department of Neurology, Columbia University, New York, NY, United States; 2 Department of Neurology, National and Kapodistrian University of Athens, Athens, Greece

**Keywords:** cognition, dementia, metabolomics, neural networks, neuroimaging

## Abstract

**Background:**

Alterations in systemic metabolism may reflect biological processes relevant to brain function and cognitive aging, yet the extent to which circulating metabolites relate to both cognitive performance and underlying neural network activity remains unclear.

**Methods:**

The current study included 112 cognitively normal participants of the Reference Abilities Neural Network study at Columbia University who had metabolomic, cognitive, and imaging data available at baseline. Cognition was measured using a neuropsychological battery including three tests for each of the four cognitive domains of memory, reasoning, processing speed, and language. Participants underwent fMRI while performing 12 tasks, 3 per domain, to derive domain-specific neural network activation pattern scores such that patterns correlated positively with behavioral performance. Plasma collected from participants were analyzed using an untargeted liquid chromatography coupled high-resolution mass spectrometry-metabolomics assay to generate metabolic profiles. Linear regression was used to explore associations between metabolite features and outcomes including cognition and neural network activation pattern scores.

**Results:**

Participants had a mean age of 56.6 years (standard deviation (SD) = 15.6), 50.0% were female, 71.4% were white, and the mean education level was 15.8 years (SD = 2.2). Of the 4,286 metabolite features analyzed, 8 were significantly associated with neural network activation pattern scores for reasoning, but no other domains after false discovery rate (FDR) adjustment. In contrast, no metabolite features were significantly associated with any cognitive domains of memory, reasoning, processing speed or language after FDR adjustment. Exploratory analyses using nominal *p*-values revealed minimal overlap in metabolite features associated with domain-specific cognitive performance and neural network measures. Six metabolite features were associated with all neural network domains, whereas only one metabolite feature was associated with all cognitive domains. Pathway-level analyses did not demonstrate consistent enrichment across domains, but suggested involvement of amino acid metabolism, lipid metabolism, energy production, cofactor and vitamin metabolism, and nitrogen handling pathways.

**Conclusion:**

Our findings suggest limited overlap in significant metabolite associations across both cognitive performance and neural network activity, which may reflect some degree of domain specificity. Neural network measures may capture aspects of underlying biological processes that are not fully reflected in cognitive test performance.

## 1 Introduction

Dementia is of significant public health concern as it is predicted to increase exponentially by the year 2050 (GBD 2019 Dementia Forecasting Collaborators, 2022). Cognitive decline and neurodegeneration preceding a dementia diagnosis begins decades before clinical symptoms become detectable (Payton et al., 2023). While domain-specific cognitive measures provide important information on functional performance, they may reflect relatively late manifestations of underlying neuropathology (Sperling et al., 2011). In contrast, neuroimaging-derived measures of brain function, including neural network activation patterns, may capture more proximal changes in brain physiology (Gonneaud et al., 2021). The integration of these complementary outcomes offers an opportunity to better characterize the biological mechanisms contributing to variation in brain function across domains.

Metabolomics provides a powerful, hypothesis-generating approach to investigate these mechanisms by comprehensively profiling small molecules that reflect downstream products of cellular processes (Schrimpe-Rutledge et al., 2016). Circulating metabolites integrate influences from genetics, environment, and lifestyle, and have been increasingly linked to cognitive function and risk of neurodegenerative disease (Machado-Fragua et al., 2022; Qiang et al., 2024; Han et al., 2011; Liu et al., 2025). Prior studies have identified associations between metabolites involved in amino acid metabolism, lipid pathways, and energy production with global cognition and dementia risk (Machado-Fragua et al., 2022; Qiang et al., 2024; Han et al., 2011; Liu et al., 2025). However, relatively few studies have examined metabolomic associations across specific cognitive domains, even fewer have evaluated whether these associations are shared relationships with functional measures of brain activity.

Neural network activation pattern scores derived from the Reference Ability Neural Network (RANN) cohort offer a domain-specific characterization of brain function based on task-related functional neuroimaging (Habeck et al., 2016). These measures quantify activation patterns corresponding to core cognitive domains, such as memory, reasoning, processing speed, and language, and may provide increased sensitivity to subtle biological variation compared to traditional cognitive test scores (Sperling et al., 2011; Habeck et al., 2018). Because functional brain network alterations may precede measurable cognitive decline and be buffered by cognitive reserve, cognitive performance and neural activation may capture distinct stages of brain aging (Sperling et al., 2011; Gonneaud et al., 2021). Examining the concordance of their significant metabolomic associations therefore provides insight into whether metabolic alterations reflect fundamental processes shared across levels of brain function or are specific to either early neural activation dysfunction or downstream cognitive expression. Investigating metabolomic correlations with both cognitive performance and neural activation patterns may therefore help disentangle shared and distinct biological pathways underlying these related, yet potentially divergent, measures of brain function. Furthermore, comparing metabolite associations across domains may reveal whether metabolic alterations meaningfully reflect common mechanisms that support multiple cognitive processes or domain-specific pathways that differentially influence memory, language, reasoning, or processing speed.

In this study, we leveraged untargeted high-resolution metabolomics to examine associations between circulating metabolite features and domain-specific cognitive performance as well as cognitive neural network activation pattern scores. Our primary objectives were to (1) characterize the extent of overlap in significant metabolite associations across cognitive domains within cognitive performance and cognitive neural network outcomes, and (2) describe the extent to which significant metabolite associations overlap between corresponding domains of cognitive performance and neural network activation. This may improve our understanding of how systemic metabolism contributes to cognitive aging and may help identify metabolomic biomarkers that are more closely linked to early neural dysfunction than conventional cognitive measures alone. By comparing metabolite associations across these complementary measures, this work provides insight into the metabolic processes that underlie early and domain-specific changes in brain function.

## 2 Materials and methods

### 2.1 Participants

The current study utilized data from participants of the Reference Abilities Neural Network (RANN) study of Columbia University. RANN is a community-based cohort study which recruited participants from the greater New York area primarily through random-market mailing. Participants were 20 to 80 years old, native English speakers, right-handed, had at least a fourth-grade reading level, were cognitively normal with a score of 135 or more on Mattis Dementia Rating Scale (Mattis, 1976), were free of other medical or psychiatric conditions that could affect cognition, and were free of functional impairment (Blessed et al., 1968). At each visit, study personnel collected demographic data, obtained blood samples, conducted imaging, including an fMRI, and administered a neuropsychological test battery. Of the 398 RANN participants, we selected 129 participants who had abundant plasma samples. After excluding 17 participants who had missing fMRI data, a total of 112 participants were included in the data analysis. The study was approved by Columbia University's Institutional Review Board. All participants provided written consent.

### 2.2 Metabolomics

A blood sample was drawn and sent to the laboratory within 2 h. Blood samples were centrifuged at 2,000 g for 15 min at room temperature and then stored at −80 °C in polypropylene cryotubes until analysis. To measure the human metabolome, plasma samples underwent untargeted liquid chromatography coupled high-resolution mass spectrometry (LC-HRMS) in the Center for Innovative Exposomics Laboratory at Columbia University. Detailed procedures of this approach has been described elsewhere (Liu et al., 2016). In brief, acetonitrile was used to extract metabolites from plasma and then injected in triplicate on two chromatography columns, including a hydrophilic interaction column under positive ionization (HIL+) and a C18 column under negative ionization (C18-). With this approach, three technical replicates per sample per column were obtained. Ions produced were analyzed in full scan mode on a Thermo Orbitrap HF Q-Exactive mass spectrometer for molecules within 85–1,250 kDa. Metabolomic data generated from this workflow were analyzed using a computational pipeline that utilized open-source software for feature detection and peak alignment, specifically apLCMS (Yu et al., 2009) and xMSanalyzer (Uppal et al., 2013). A table of features including data on mass-to-charge (M/Z) ratio, retention time, and abundance/intensity of each ion for each sample was formed and then corrected for batch effects using ComBat, which employs an empirical Bayesian framework to adjust for known batch effects across sample runs (Leek et al., 2012).

Each unique M/Z and retention time combination is referred to as a unique metabolite feature. Prior to analysis, data was preprocessed as described in Figure 1. A total of 7,327 metabolite features were identified using the HIL+ column and 6,806 features using the C18- column. Values with zero-intensity were considered below the detection limit. From both columns, a total of 4,286 metabolite features detected in at least 70% of the samples were included in the analysis. For metabolite features that met the inclusion criteria, values below the detectable limit were imputed with half the minimum intensity observed for each metabolic feature. Finally, we log10 transformed intensity values for each metabolite feature and auto-scaled prior to analysis to normalize and standardize the data. As an exploratory analysis, PCA was performed to characterize the correlation structure and dimensionality of the metabolomic data, with the proportion of variance explained by each component visualized using a scree plot; principal components were not used in subsequent outcome analyses.

**Figure 1:**
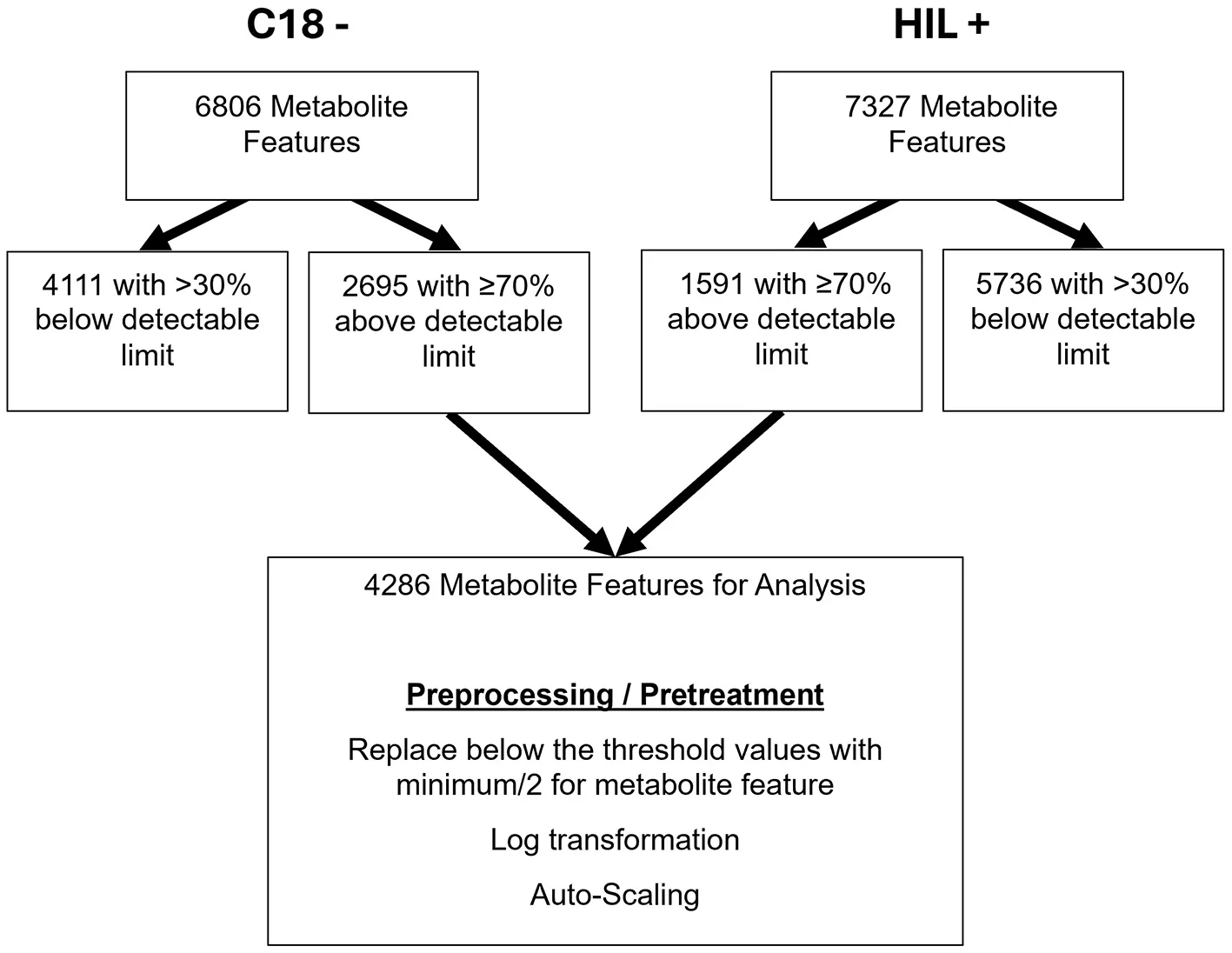
Metabolite feature selection for analysis.

### 2.3 Neural network activation patterns scores

Development of neural activation pattern scores, including in-scanner tests and procedures, fMRI data acquisition, fMRI data processing, and score derivation have been described in detail elsewhere (Habeck et al., 2016). In brief, fMRI data was obtained while participants completed 12 in-scanner cognitive tasks, three for each of the four domains, including episodic memory, fluid reasoning, perceptual processing speed, and language (Stern et al., 2014). Tasks were completed in a fixed order over two scanning sessions, ~2 h each in length, memory and fluid reasoning tests in one session, and language and speed in the other. However, the order of the two sessions was counterbalanced across participants. Prior to each in-scanner session, participants completed a training session on a laptop computer to familiarize themselves with the tasks. The memory domain included tests of logical memory, word order recognition, and paired associates. Fluid reasoning included tests of paper folding, matrix reasoning, and letter sets. Speed tests included digit symbol, letter comparison and pattern comparison. Finally, language included synonyms, antonyms, and picture naming. All tasks, except for picture naming, required responses via button press, whereas picture naming involved a vocal response.

Images were acquired using a 3T Philips Achieva Magnet. At each session, participant positioning was verified with a scout T1-weighted image. High-resolution structural images were obtained using a T1-weighted MPRAGE sequence (TE/TR = 3/6.5 ms; flip angle = 8°; in-plane resolution = 256 × 256; field of view = 25.4 × 25.4 cm), yielding 165–180 axial slices with a slice thickness of 1 mm and no interslice gap. All structural scans used a 240 mm field of view. Functional images were acquired using a gradient-echo EPI sequence with the following parameters: TE/TR = 20/2,000 ms; flip angle = 72°; in-plane resolution = 112 × 112; slice thickness = 3 mm with no gap; and 41 axial slices.

Each individual's 12 fMRI scans were pre-processed using the FSL software package (Smith et al., 2004), and went through the following steps: (1) computation of a within-subject histogram for each subject volume to identify noise (FEAT); (2) correction for subject-motion (MCFLIRT); (3) correction for slice-timing; (4) brain-mask creation from first volume in subject's fMRI data; (5) high-pass filtering (T = 128 sec); (6) pre-whitening; (7) General-Linear-Model (GLM) estimation with equally temporally filtered regressors and double-gamma hemodynamic response functions; (8) registration of functional and structural images with subsequent normalization into MNI space (FNIRT).

Task-related brain activity was first quantified by fitting a voxelwise general linear model (GLM) separately for each participant and each task, yielding a task-specific activation (β) map that reflects the contrast between task engagement and baseline or control conditions. We then computed reference-domain activation patterns in a simplified version of our 2018 analysis (Habeck et al., 2018), which was performed on an updated sample of 322 participants (from the total sample of 398 people) who possessed maps for all 12 tasks. We averaged both neural activation and task performance data of the three tasks pertaining to one domain into a mean activation map and one performance value, respectively, and performed the Scaled Subprofile Modeling (SSM-PCA) (Moeller and Strother, 1991; Habeck and Stern, 2010).

SSM-PCA derives an activation pattern whose subject scores best predict the cognitive performance outcome. The pattern score served as an outcome of interest for the metabolomics data. We performed four SSM-PCA analyses, one for each reference domain. Contrary to our 2018 analysis, we did not impose the constraint of uniqueness of each reference domain; this is conceptually apt since some common processing can be expected for all tasks. Further, analyzing each cognitive domain separately substantially simplified the analytical approach. More information about the derived activation patterns with topographic listings can be found in Appendix A of the supplemental methods (Supplementary Figure 1 and Supplementary Tables 1–4).

### 2.4 Cognition

As part of the visit but conducted in a separate session than fMRI data collection, participants completed a neuropsychological battery consisting of 12 tests, with three tests per cognitive domain. The tests in this battery, however, differed from those administered during the in-scanner assessment. Memory was assessed using the Selective Reminding Test (SRT) Long-Term Storage, SRT Continuous Long-Term Retrieval, and SRT Total Words Recalled on the Last Trial (Buschke and Fuld, 1974). Reasoning was evaluated using the WAIS-III Block Design and Letter–Number Sequencing tasks. Processing speed was measured with the WAIS-III Digit Symbol task, Trail Making Test Part A, and the Stroop Color Naming Test. Language ability was assessed using the WAIS-III Vocabulary subtest, the Wechsler Test of Adult Reading, and the American National Adult Reading Test. A summary score for each domain was calculated using principal axis factor analysis and then z-scored so that each domain-specific cognitive score had a mean of 0 and standard deviation of 1 for the entire cohort. With this approach, higher scores indicate better cognitive performance.

### 2.5 Statistical analyses

#### 2.5.1 Metabolome-wide association with neural network and cognition

Metabolome-wide association study (MWAS) analyses were conducted independently for each outcome, four neural network-derived domains and four cognitive domains, yielding a total of 8 MWAS analyses. With this framework, a statistical model was fit for each metabolite feature adjusting for age and sex for neural network analyses and age, sex, and education for cognition analyses. For our primary analysis, we accounted for multiple comparisons by applying an FDR of 5% and q-values were estimated using the Benjamini-Hochberg method. As few to no metabolite features remained significant for each MWAS after adjustment for multiple comparisons, an exploratory analysis using a nominal *p*-value cutoff of 0.05 was conducted. As a sensitivity analysis, metabolites with 25–30% of values below the limit of detection (LOD) were reanalyzed after treating values below the LOD as missing. Primary models were repeated using the resulting available-case samples. Robustness was assessed by calculating the correlation between beta estimates from the primary and sensitivity analyses among metabolites significant in the primary analysis. As a complementary analysis, weighted gene co-expression network analysis (WGCNA) was used to identify modules of correlated metabolites. Module eigengenes, representing the first principal component of the metabolites within the module, were then evaluated for associations with cognitive and functional neural network outcomes using regression models adjusted for the same covariates as the primary analyses.

#### 2.5.2 Metabolite annotation

In order to gain an understanding of what metabolites may be represented, we assigned metabolite annotations using an internal library created using chemical standards run on the platform to obtain m/z and retention time information to support high-confidence metabolite annotations, and by mapping to the Human Metabolome Database (HMDB; Wishart et al., 2022) using the R package xMSannotator (version 1.3.2; Uppal et al., 2017). Of the 4,286 metabolite features analyzed, 2,971 (69%) were mapped back to putative metabolites. If a metabolite feature was matched to multiple candidate metabolites, we selected the annotation with the highest confidence score assigned by xMSannotator for interpretation purposes (Schymanski et al., 2014). In cases where multiple annotations shared the same confidence score, we used the annotation with the smallest difference between expected and observed mass. For metabolite features where both the confidence score and delta PPM were identical across annotations, we reported the multiple possible identities, as we could not reasonably identify a unique annotation. If a feature was not mapped back to a database, we considered this metabolite feature as “unknown” (31%).

#### 2.5.3 Exploratory pathway analysis

Given that individual metabolites may interact and act together within the same biological pathways, and that their collective effects may be relevant to cognition and neural network outcomes even when the effect of each individual metabolite is small, we performed pathway analysis to explore the biological importance of metabolic features associated with the outcomes, using the functional analysis module in MetaboAnalyst (version 5.0), a web-based platform for comprehensive metabolomics analysis (Pang et al., 2022). MWAS results of both chromatographic columns were incorporated. Up to 86 pathways were examined in MetaboAnalyst. As this analysis was exploratory, a nominal *p*-value threshold (<0.05 or <0.10) was selected to ensure inclusion of at least 10% of metabolite features for each outcome in pathway enrichment analyses using the mummichog algorithm with the human MFN reference database (Li et al., 2013). Nominal *p*-values were used for this analysis, as few to no FDR adjusted *p*-values met these criteria when examining outcomes. Pathway-level significance was evaluated using Fisher's exact test, and results with *p*-values <0.05 are reported and interpreted as exploratory.

## 3 Results

### 3.1 Study sample

Of the 398 RANN participants, 112 had complete metabolomic, cognitive, and neural network data available for analysis. Participants included had a mean age of 56.6 years, a mean education level 15.8 years, and were predominantly white (71.4%) and non-Hispanic (82.1%; Table 1). Women and men each represented half of the analytic sample. Compared to those excluded, the 112 RANN participants in this study were older (*p* = 0.0104) and had higher language neural network activation patterns scores (*p* = 0.0006) and higher cognition scores for reasoning (*p* = 0.0267) and language (*p* = 0.0013). Principal component analysis revealed no dominant underlying structure in the metabolomic dataset. Although the first 3 principal components explained the greatest proportion of variance (PC1 = 13%, PC2 = 8%, PC3 = 5%), the scree plot (Supplementary Figure 2) exhibited a gradual decline without a pronounced elbow, suggesting that variability is distributed across many correlated metabolite features rather than captured by a small number of latent dimensions. In the sensitivity analysis treating metabolite values below the LOD as missing, beta estimates were generally consistent with those from the primary analysis, with correlations ranging from 0.66 to 0.83 across outcomes, supporting the robustness of the observed associations to the handling of values below the LOD (Supplementary Table 5).

**Table 1:** Study sample characteristics.

	Overall RANN Cohort (*N* = 398)		Excluded Participants (*N* = 286)		Neural network metabolomics subgroup (*N* = 112)		*P*-value^*^
Characteristic	Mean or *N*	SD or %	Mean or *N*	SD or %	Mean or *N*	SD or %	
Age (years)	53.1	17.0	51.7	17.3	56.6	15.6	0.0104
Education (years)	15.8	2.4	18.8	2.4	15.8	2.2	0.8664
Sex							0.1852
Female	220	55.3%	164	57.3%	56	50.0%	
Male	178	44.7%	122	42.7%	56	50.0%	
Race							0.1796
Asian	20	5.0%	16	5.6%	4	3.6%	
Black	94	23.6%	71	24.8%	23	20.5%	
White	247	62.1%	167	58.4%	80	71.4%	
Other	37	9.3%	32	11.2%	5	4.5%	
Ethnicity							0.8184
Hispanic	72	81.7%	52	18.2%	20	17.9%	
Not Hispanic	325	18.1%	233	81.5%	92	82.1%	
Unknown	1	0.3%	1	0.4%	0	0.0%	
Cognition Z-scores							
Memory	0.024	0.932	0.017	0.970	0.040	0.836	0.8257
Reason	0.023	0.802	−0.035	0.791	0.164	0.814	0.0267
Speed	−0.006	0.817	0.020	0.810	−0.069	0.835	0.3357
Language	−0.042	0.943	−0.141	0.981	0.200	0.795	0.0013
Neural network activation scores							
Memory	0.008	0.766	0.012	0.726	0.002	0.836	0.9080
Reason	0.005	0.861	−0.012	0.845	0.034	0.892	0.6476
Speed	0.000	0.860	0.064	0.845	−0.116	0.879	0.0759
Language	0.015	0.846	−0.107	0.841	0.235	0.813	0.0006

^*^Tests (t-test for continuous variables and chi-squared test for categorical) comparing those in the neural network metabolomics subgroup and those excluded.

RANN, reference ability neural network; *N*, number; SD, standard deviation.

### 3.2 Primary analysis: associated metabolite features after FDR adjustment

After FDR adjustment for multiple comparisons, no metabolite features were associated with any of the four cognitive domains, nor the neural network activation pattern scores for memory, speed, or language. However, 8 metabolite features were significantly associated with the neural network activation pattern for reasoning, including 1 metabolite feature from the C18- column, and 7 metabolite features from the HIL+ column. Of the 8 metabolite features, 5 were annotated, including three annotated as creatinine, one as a sphingomyelin, and one matched to multiple metabolites including phosphatidylcholines and phosphatidylethanolamines (Table 2). WGCNA identified 22 modules of correlated metabolites. No associations between module eigengenes and study outcomes were statistically significant after FDR adjustment.

**Table 2:** Metabolite features and their annotations associated with reasoning neural network activation pattern scores after adjustment for multiple comparisons.

M/Z	Time (s)	Annotation	Formula	Pathway/source	ESI	Estimate (95 Confidence Interval)
334.9485	30.8	Unknown	-	-	-	−0.374 (−0.542, −0.205)
210.8874	53.1	Unknown	-	-	+	−0.325 (−0.476, −0.174)
564.6744	51.9	Unknown	-	-	+	−0.299 (−0.444, −0.154)
114.0664	34.3	Creatinine	C4H7N3O	Creatine metabolism; urea cycle	+	−0.312 (−0.456, 0.168)
675.5435	31.0	SM(d18:1/14:0)	C37H75N2O6P	Sphingolipid metabolism; membrane structure; myelin biology	+	0.306 (0.161, 0.451)
706.5383	28.6	PC(16:0/14:0) PC(14:0/16:0) PC(15:0/15:0) PE (18:0/15:0) PE(15:0/18:0) PE-NMe (16:0/16:0)	C38H76NO8P	Glycerophospholipid metabolism; membrane lipid metabolism	+	0.322 (0.185, 0.460)
115.0632	31.1	Creatinine	C4H7N3O	Creatine metabolism; urea cycle	+	−0.295 (−0.437, −0.152)
115.0696	31.7	Creatinine	C4H7N3O	Creatine metabolism; urea cycle	+	−0.294 (−0.433, −0.156)

### 3.3 Exploratory analysis: outcome-specific associations

Using the MWAS approach and a nominal *p*-value cut-off of 0.05, 1,199 metabolite features were associated with one or more neural network activation pattern scores (Figure 2), of which 6 metabolite features were associated with all 4 domains (Table 3). Of the 6 associated with all domains, 5 were annotated. However, 3 have a confidence level 5, suggesting little confidence in annotation. The two matched with a confidence level of 3 or above had multiple annotations (Supplementary Table 6).

**Figure 2:**
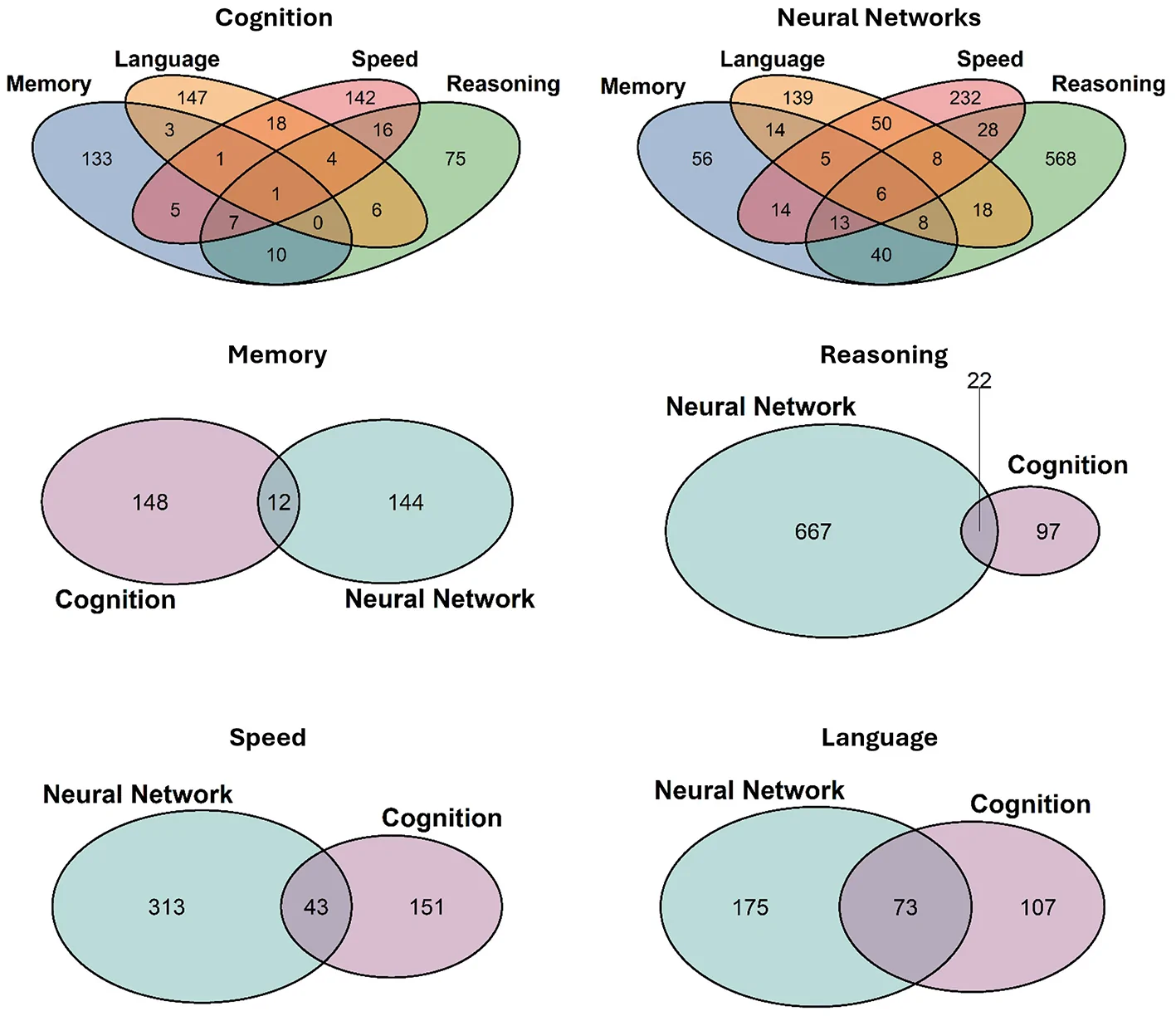
Overlap of metabolites significantly associated with domain-specific cognition and corresponding neural network activation after adjusting for age, sex, and education.

**Table 3:** Distribution of metabolite features by number of associated domain.

Number of domains	Metabolite features associated	Percent
Cognition		
0	3,718	86.8%
1	497	11.6%
2	58	1.4%
3	12	0.3%
4	1	0.02%
Neural network activation		
0	3,087	72.0%
1	995	23.2%
2	164	3.8%
3	34	0.8%
4	6	0.1%

A total of 568 metabolite features were associated with at least one or more cognition domains (Figure 2). Only one was associated with all 4 cognitive domains and was putatively annotated as multiple metabolites representing methylated inositol isomers (Supplementary Table 6).

### 3.4 Exploratory analysis: shared metabolites between cognition and neural network

Figure 2 displays the overlap between metabolites significantly associated with domain-specific neural network activation and corresponding cognition. Counts are shown for metabolites that are significant in neural network activation only, cognition only, or both. In total, 12, 22, 43, and 73 metabolites were associated with same-domain neural network activation and cognition for memory, reasoning, speed, and language, respectively. No metabolite feature was associated with all neural network domains and cognitive domains. Of the six metabolite features associated with all four neural network domains, two were also associated with cognitive domains of processing speed and language, while an additional two were associated exclusively with language.

### 3.5 FDR adjusted pathway analysis

Table 4 summarizes the enriched pathways identified for each domain across neural network activation pattern and cognition. After FDR adjustment, only a few pathways remained significant, including arginine and proline metabolism for the memory neural network activation pattern, as well as *de novo* fatty acid biosynthesis and fatty acid oxidation for the reasoning neural network activation pattern. No pathways for cognition domains remained significantly enriched after FDR adjustment. Pathway analyses are presented in more detail in supplemental results Supplementary Tables 7–10 and Supplementary Figures 3–5.

**Table 4:** Pathway analysis of metabolites significantly associated with domain-specific cognition and neural networks.

	Memory		Reason		Speed		Language	
Pathway	Cognition	Neural network	Cognition	Neural network	Cognition	Neural network	Cognition	Neural network
Alanine and aspartate metabolism					^*^	^*^		
Arginine and proline metabolism		^**^	^*^		^*^			
Ascorbate (vitamin C) and aldarate metabolism					^*^			
Aspartate and asparagine metabolism		^*^						
Beta-Alanine metabolism	^*^	^*^						
Biopterin metabolism						^*^		
Butanoate metabolism	^*^	^*^						
*De novo* fatty acid biosynthesis				^**^				
Di-unsaturated fatty acid beta-oxidation				^*^				
Fatty acid activation				^**^				
Fatty acid oxidation				^**^				
Fatty acid oxidation, peroxisome				^*^				
Glycine, serine, alanine and threonine metabolism		^*^						
Glyoxylate and dicarboxylate metabolism								
Histidine metabolism	^*^						^*^	
Lipoate metabolism		^*^						
Lysine metabolism	^*^	^*^						
Mono-unsaturated fatty acid beta-oxidation				^*^				
Nitrogen metabolism	^*^				^*^			
Omega-3 fatty acid metabolism				^*^				
Polyunsaturated fatty acid biosynthesis				^*^				
Propanoate metabolism		^*^						
Pyrimidine metabolism								^*^
Pyruvate Metabolism					^*^			
Selenoamino acid metabolism						^*^		
Valine, leucine and isoleucine degradation	^*^	^*^				^*^		
Vitamin H (biotin) metabolism						^*^		
Urea cycle/amino group metabolism	^*^	^*^			^*^			

^*^ Nominal *p*-value <0.05.

^**^ FDR *p*-value <0.05. Light green indicates p-value reaches nominal significance, dark green indicates p-value reaches significance after FDR adjustment.

### 3.6 Exploratory pathway analysis

When exploring nominal associations, the memory domain had the largest variety in affected pathways, with 10 for neural networks and 7 involved for cognition. In addition, the memory domain showed the greatest overlap, sharing 5 enriched pathways between neural networks and cognition, including beta-alanine metabolism, butanoate metabolism, lysine metabolism, valine, leucine and isoleucine degradation, and urea cycle/amino group metabolism. There were 0, 1, and 0 overlapping pathways for reasoning, speed, and language domains, respectively.

## 4 Discussion

In this cross-sectional study of 112 participants, we found few metabolite features were significantly associated with the reasoning domain for neural network activation pattern score while no metabolite features were significantly associated with other cognitive or neural network outcomes after adjusting for multiple comparisons. In exploratory analyses without adjusting for multiple comparisons, hundreds of metabolites were found to be associated with these outcomes but findings demonstrated minimal overlap in these associations across different domains, potentially indicating a degree of domain specificity; and minimal overlap between cognition and neural network outcomes of the same domain, indicating outcome specificity.

### 4.1 Primary finding on the associated metabolite features after FDR adjustment

Our findings suggest that the reasoning neural network domain is potentially the most metabolically demanding and thus maybe more sensitive to metabolic perturbations (Bo et al., 2025; Crossley et al., 2014; Jamadar et al., 2025). For three metabolite features annotated as creatinine, a nitrogen-containing byproduct of creatine metabolism (Wyss and Kaddurah-Daouk, 2000), higher metabolite levels were consistently found to be significantly associated with a lower reasoning neural network activation pattern scores in our primary analysis adjusting for multiple comparisons.

However, the relationship between plasma creatinine and energetics in the brain is somewhat tenuous. Oral creatine supplementation has been associated with an increase in levels of creatine in the brain (Smith et al., 2025; Lyoo et al., 2003). However, while creatine may be converted to creatinine within brain cells to generate additional energy, particularly when mitochondria are not functioning optimally, this would have little influence on creatinine blood levels (Forbes et al., 2022). Plasma creatinine levels may be more indicative of kidney function. As plasma creatinine increases, this may suggest the kidneys may not be properly filtering the blood (Inker et al., 2021). A reduction in kidney performance has been associated with cognitive decline and dementia (Kelly et al., 2024; Wang et al., 2024; Tang et al., 2022). While chronic kidney disease and cognitive decline have overlapping risk factors, such as blood pressure and vascular diseases (Hsu et al., 2005; Walker et al., 2019; Guzik et al., 2024), it is hypothesized that impaired kidney function can contribute to cognitive decline through mechanisms including chronic inflammation, uremic neurotoxicity, and vascular dysfunction (Kelly et al., 2024; Andrews et al., 2025). Importantly, there may be additional causes that contribute to high creatinine levels that may be confounding relationships on neural network activation, including greater muscle mass, higher meat intake, or medication use (Antonio et al., 2021).

Additional metabolite features positively associated with the reasoning neural network activation pattern score in our primary analysis also included those related to lipid metabolism. These metabolite features were annotated as phosphatidylcholines, phosphatidylethanolamines and a sphingomyelin. Results have been mixed when examining the relationship between these individual metabolites and brain health (Han et al., 2011; Moseholm et al., 2024; Lefèvre-Arbogast et al., 2021; Jiang et al., 2019). Mixed results may indicate disruption in these pathways may be the problem rather than altered levels of individual lipid species. Although these phospholipids do not freely cross the blood-brain barrier, plasma phospholipids may act as markers of a metabolic state in the brain (Borkowski et al., 2023; Iriondo et al., 2021). Given their central role in membrane structure and synaptic signaling (Wood, 2012), these phospholipids may facilitate the expression of activation patterns associated with better task performance through effects on membrane integrity, signaling processes, and resistance to oxidative stress.

### 4.2 Overlapping metabolites across domains

In our exploratory analysis using a nominal *p*-values, one of the 6 metabolite features associated with all four neural network activation patterns was annotated as several lipids. These annotated metabolites represent ether-linked phosphatidylcholines, including several plasmalogen species, which are key components of cellular membranes (Dean and Lodhi, 2018). Ether lipids play important roles in membrane organization, lipid raft formation, vesicular trafficking, and antioxidant defense, and are particularly enriched in tissues with high membrane turnover such as the brain (Dean and Lodhi, 2018). Although circulating ether phosphatidylcholines do not directly reflect brain lipid composition due to the blood–brain barrier, they may index systemic ether lipid metabolism relevant to neural function (Ranasinghe and Hla, 2025).

Additionally, desmosterol and 7-dehydrocholesterol, direct precursors to cholesterol, appear to be associated with all four neural networks. Increased desmosterol and 7-dehydrocholesterol may be suggestive of a disrupted cholesterol homeostasis (Brown et al., 2014). Though plasma cholesterol and its precursors are unable to readily pass through the blood brain barrier, prior studies have shown plasma desmosterol/cholesterol ratios correlate with CSF desmosterol/cholesterol ratios (Sato et al., 2012). Thus, the association of greater plasma levels of these cholesterol precursors with greater activation patterns across domains may reflect increased availability of metabolic resources influencing brain function. Age-related reductions in cholesterol levels in cell membranes lead to synaptic loss and impaired synaptic plasticity (Martin et al., 2008; Sodero et al., 2011). Alternatively, higher plasma levels of desmosterol or 7-dehydrocholesterol could influence inflammation, vascular health or hormone signaling that can subsequently influence brain function through broader metabolic pathways (Zhang et al., 2021; Zerenturk et al., 2013).

Three of the 6 metabolic features were putatively annotated as isepamacin, fluticasone and ganoderic acid H, which are not endogenous in humans nor likely to be influenced by diet in our population to achieve a detectable measurement in greater than 70% of our sample (Cui et al., 2026; Stokes et al., 2014; Ansari et al., 2022). Additionally, these were putatively annotated with a confidence level of 5, suggesting very low confidence, and are likely misannotated. However, notably, these 3 metabolic features were also the only features to also be significantly associated with at least 2 cognitive domains (speed and language).

Interestingly, the only one feature positively associated with all cognitive domains was annotated to multiple methylated inositol isomers (cyclitols; e.g., pinitol/quebrachitol-type compounds) suggesting a broad, domain-general relationship with cognitive function. Although the specific isomer cannot be distinguished in untargeted data, these compounds belong to the inositol metabolite family, which plays a central role in phosphoinositide signaling, a pathway critical for synaptic transmission and neuronal plasticity (Pitt et al., 2013). In addition, methylated inositols have been linked to insulin signaling and metabolic regulation, processes increasingly implicated in global cognitive performance and neurodegeneration (Pitt et al., 2013; Rönnemaa et al., 2008; Arvanitakis et al., 2024). The consistency of associations across diverse cognitive domains supports the interpretation that this signal reflects systemic metabolic pathways influencing overall brain function rather than domain-specific effects. Methyl inositols can also be obtained from dietary sources, particularly legumes, and may therefore represent potential mediators linking healthy dietary patterns to reduced dementia risk (Siracusa et al., 2022).

There was limited overlap in significant metabolite features between domains among either our two outcomes of cognition and neural network activation. This highlights the potential for domain specificity and that metabolic signatures identified for one cognitive domain may not necessarily generalize to others. Our pathway analysis further supports this, as no pathway was significantly enriched for all domains for either outcome. However, some themes do emerge. Among all four domains, the memory domain for both neural networks and cognition had the largest variety in affected pathways and the greatest overlap between the two outcomes. The greater overlap observed in the memory domain may reflect tighter coupling between metabolic processes, neural network activity, and cognitive performance in memory-related systems. Memory relies on metabolically demanding hippocampal networks that are particularly vulnerable to early neurodegenerative changes, making it potentially sensitive to perturbations in a broad range of metabolic pathways compared to other domains (Rao et al., 2022).

In contrast, pathways involved in the reasoning domain had no overlap between neural networks activation and cognition outcomes. However, reasoning neural network activation had the strongest and most consistent signal, with enrichment of pathways involved in lipid metabolism, specifically how the cell makes, activates, and breaks down fatty acids for energy or storage. These findings suggest that the neural network for reasoning may be particularly sensitive specifically to disruptions in metabolic processes that support high energetic demand and cellular maintenance, aligning with the greater computational complexity and metabolic requirements of higher-order cognitive functions.

Other domains showed comparatively few enriched pathways involved. Speed was linked to a small number of pathways, including biopterin and pyruvate metabolism, while language demonstrated minimal metabolic signal overall, consistent with its characterization as a more stable, crystallized cognitive domain (Cattell, 1963). Collectively, these findings indicate that systemic metabolic perturbations do not uniformly affect cognitive domains, but instead preferentially impact specific neural systems, with memory and reasoning networks appearing especially vulnerable. This domain-specific metabolic sensitivity may provide insight into early biological mechanisms underlying cognitive decline and highlights the importance of examining neural phenotypes alongside traditional cognitive measures.

### 4.3 Shared metabolites between cognition and neural network of the same domain

There were certain metabolite features associated with both cognitive scores and neural network activity of the same domain, strongly supporting the role of these metabolites in the corresponding domain. However, as most of the metabolites were selectively associated with either neural network activation patterns or cognition, results may suggest different metabolites may be involved in distinct, albeit related, aspects of brain function. For example, neural network activation measures BOLD, potentially reflecting physiological processes that are more closely related with metabolites in the blood than cognitive test performance. Alternatively, the different cognitive tests used in the scanner and outside of the scanner may also explain partially the discrepancy of the domain-related metabolites. Consistent with the individual metabolites' findings, few overlapping pathways across domains or outcomes were identified, with the potential exception of the memory domain which had 5 shared enriched pathways between outcomes.

### 4.4 Strengths and limitations

This study has several important strengths and limitations. A key strength is the integration of high-resolution metabolomics with neural network activation pattern scores derived from Reference Ability Neural Network (RANN) analyses, providing a unique opportunity to examine the biological underpinnings of domain-specific brain function. The use of RANN-derived measures enables a sensitive, domain-targeted assessment of neural activation across cognitive systems, enhancing the ability to detect metabolite–brain relationships that may not be captured by traditional cognitive measures. Additionally, the use of untargeted metabolomics allows for a comprehensive, hypothesis-free assessment of a broad range of metabolic pathways, facilitating the identification of novel biological signals. Finally, this study was conducted in cognitively normal adults spanning ages 20–80, enabling the investigation of biological mechanisms that may contribute to cognitive decline decades before clinical symptoms emerge.

However, several limitations should be considered. The relatively small sample size (n=112) limits statistical power, particularly in the context of high-dimensional metabolomics data. While our primary analysis did adjust for multiple comparisons, few associations remained significant after correction, reducing our ability to draw definitive conclusions and increasing the potential of overlooking meaningful effects. Thus, we conducted an exploratory analysis using nominal *p*-values, however this has increased likelihood of false positives and must be interpreted with caution. Additionally, participants with available neural network data who were included in the analytic sample differed from the overall cohort in age and several cognitive and neural network measures, raising the possibility of selection bias and potentially limiting the generalizability of these findings to the broader cohort. The neural network activation measures were summarized as domain-specific network scores, which do not capture the functional heterogeneity within individual networks. Consequently, metabolite associations specific to distinct regions or subnetworks may have been attenuated or obscured. Further, some metabolite annotations are putative and may be subject to misclassification without confirmatory MS/MS validation. Also, circulating plasma metabolites may not directly reflect central nervous system processes, limiting inference regarding brain-specific mechanisms. Finally, residual confounding by factors such as diet, kidney function, and medication use cannot be ruled out.

### 4.5 Conclusions

In conclusion, our findings suggest that circulating metabolomic profiles show limited robust associations with domain-specific cognitive performance but may demonstrate more targeted relationships with neural network activation, particularly within the reasoning domain. The minimal overlap in metabolite features across cognitive and neural activation outcomes, as well as across domains, underscores the heterogeneity of the biological processes underlying different aspects of brain function. These results further suggest that neural network activation patterns may capture more proximal or sensitive metabolic signatures compared to traditional cognitive measures. While pathway analyses pointed to broad metabolic processes, including amino acid and lipid metabolism, energy production, cofactor and vitamin metabolism, and nitrogen handling, these signals were not consistent across domains, reinforcing the complexity and the potential for domain specificity of these relationships. Given the exploratory nature and limited sample size, these findings should be interpreted cautiously but highlight the potential of integrating metabolomics with neuroimaging-derived measures to uncover early and potentially domain-specific mechanisms of brain function. Future studies in larger, well-characterized cohorts with longitudinal designs are needed to validate these associations, clarify their relevance for neurodegeneration, and investigate metabolomic relationships with finer-grained functional subnetworks or region-specific activation patterns.

## Data Availability

The datasets analyzed in this study are not publicly available due to participant confidentiality and institutional data-sharing restrictions. De-identified data may be made available upon reasonable request, subject to applicable institutional approvals and data-sharing agreements. Requests to access the datasets should be directed to the corresponding author.
