# Supplementary material for: Domain-specific associations of metabolomic features with cognition and neural networks among cognitively normal adults
Source: Front Neurosci. 2026 Oct 20;20:1898605. doi: 10.3389/fnins.2026.1898605 (PMC13727718; doi:10.3389/fnins.2026.1898605)
Supplement: Supplementary file 1 [file Data_Sheet_1.pdf]

Appendix A. Additional Methods for Neural Network Activation Pattern Scores

We performed an analysis approach that was an updated version of the approaches offered in previous analyses (1, 2). Preprocessing and first-level time series detailed were the same as shown in (1). These previous studies incorporated the constraint of uniqueness for each reference-domain activation pattern: this was overly stringent and necessitated the combined analysis of *all* task data with extensive non-parametric inference. Conceptually, this was also overly rigorous since it systematically stripped out task-invariant processes that might be common to all tasks. We thus simplified our analyses and performed the long-standing Scaled Subprofile Model (SSM) (3-6) which has a long history of successful application in our group.

A subset of 322 participants aged 20 to 80 had task-activation for all 12 tasks. Task map and cognitive performance data were averaged for the 3 tasks pertaining to each cognitive reference domain. The performance measure for the speed tasks was inverted reaction time; this was done such that the valence of the behavioral variable was fixed and higher values implied better performance. Likewise, positive and negative loadings meant activation was *positively* and *negatively* related to task performance, respectively.

For any reference domain, the neural activation data could be written as **X** with format [X]= voxels x participants. Performance data can be written as **y** with [**y**]**=** participants x 1. The pattern derivation can be written as a pseudo function:

$$\mathbf{v}=\mathrm{SSM}(\mathbf{X},\mathbf{y})$$

where **v** is a pattern, i.e., a column vectors of loadings. The pseudo-function incorporates the PCA-step with brain behavioral fitting and the selection of the best-fitting PC-set with the AIC criterion (7, 8).

A bootstrap resampling procedure was conducted, and neural and behavioral data were resampled with replacement was conducted 500 times, repeating the analysis while using the PC-set determined form the point estimate, generating an ensemble of 500 bootstrap images (1). A Z-map was computed as the point estimate divided by the bootstrap variability around it for each voxel-loading. Absolute Z-values of $|Z|>2$ were taken as robust loadings for visualization.

The results are shown in the supplemental figure S1. All reference domains produced highly significant patterns with robust loadings.

Figure S1. Domain-specific neural network activation pattern loadings.

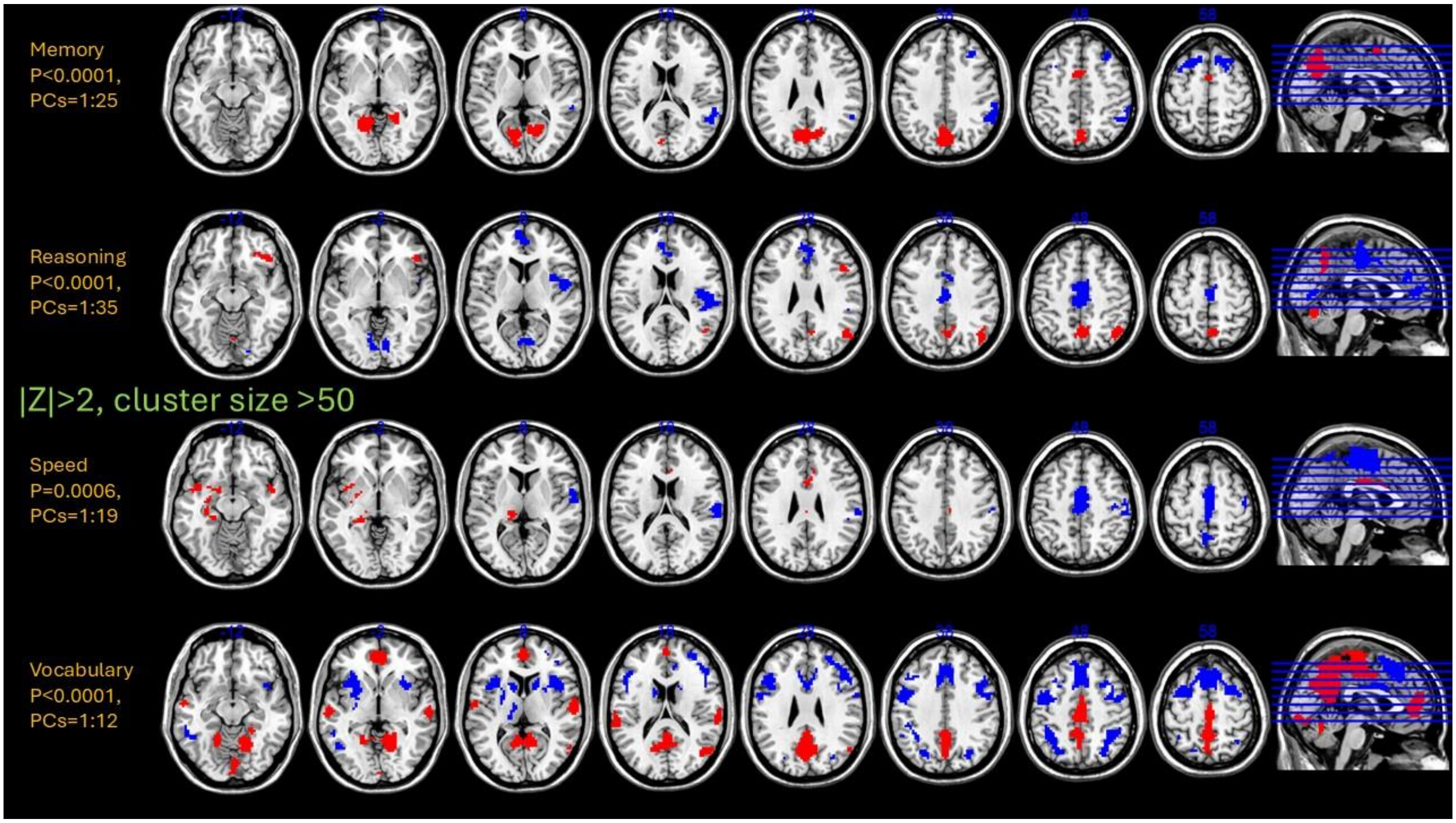

Table S1. Loadings for memory activation pattern scores.

Loading Direction	X	Y	Z	CS	bootstrap-Z	AAL_label
Positive	-3	-78	39	711	3.4099	Cuneus_L
Positive	3	-72	30	711	3.3236	Cuneus_R
Positive	-9	-72	27	711	3.1056	Cuneus_L
Positive	-12	-69	6	711	3.0372	Lingual_L
Positive	18	-63	6	179	2.8221	Calcarine_R
Positive	6	-57	3	179	2.7331	Lingual_R
Positive	-9	-54	0	711	2.6542	Lingual_L
Positive	-6	6	48	50	2.6445	Supp_Motor_Area_L
Positive	12	-69	27	711	2.6338	Cuneus_R
Positive	-21	-57	-3	711	2.5696	Lingual_L
Positive	21	-48	-3	179	2.5614	Lingual_R
Positive	-9	-81	12	711	2.4957	Calcarine_L
Positive	9	-48	0	179	2.4743	Lingual_R
Positive	-3	-63	39	711	2.4082	Precuneus_L
Positive	-9	-45	3	711	2.2936	Lingual_L
Positive	0	-57	30	711	2.1483	Precuneus_L
Negative	-15	21	63	108	-3.0803	Frontal_Sup_L
Negative	60	-42	45	129	-3.0580	SupraMarginal_R
Negative	60	-51	42	129	-3.0229	Parietal_Inf_R
Negative	9	24	60	175	-3.0032	Supp_Motor_Area_R
Negative	33	33	42	175	-2.8369	Frontal_Mid_R
Negative	27	24	42	175	-2.7107	Frontal_Mid_R
Negative	15	15	63	175	-2.6785	Supp_Motor_Area_R
Negative	21	21	54	175	-2.6183	Frontal_Sup_R
Negative	-27	18	57	108	-2.5755	Frontal_Mid_L
Negative	48	-51	48	129	-2.5597	Parietal_Inf_R
Negative	60	-45	21	89	-2.5484	Temporal_Sup_R
Negative	48	-51	15	89	-2.3728	Temporal_Mid_R
Negative	60	-36	9	89	-2.1984	Temporal_Sup_R
Negative	-36	9	54	108	-2.1758	Frontal_Mid_L
Negative	48	-48	24	89	-2.1284	Angular_R
Negative	63	-54	24	89	-2.1146	Temporal_Sup_R

CS: Cluster Size; AAL: Automated Anatomical Labeling

Table S2. Loadings for reasoning activation pattern scores.

Loading Direction	X	Y	Z	CS	bootstrap-Z	AAL_label
Positive	9	-63	51	169	3.2435	Precuneus_R
Positive	48	30	-9	104	2.9970	Frontal_Inf_Orb_R
Positive	36	30	-18	104	2.9969	Frontal_Inf_Orb_R
Positive	42	-63	51	193	2.7291	Angular_R
Positive	48	-72	36	193	2.7146	Angular_R
Positive	3	-75	-18	223	2.6859	Vermis_7
Positive	51	-63	27	193	2.5921	Angular_R
Positive	24	36	-15	104	2.5707	Frontal_Sup_Orb_R
Positive	15	-63	-27	223	2.5514	Cerebellum_6_R
Positive	51	-60	48	193	2.4850	Angular_R
Positive	6	-60	33	169	2.4238	Precuneus_R
Positive	45	-54	42	193	2.3477	Parietal_Inf_R
Positive	-6	-72	-24	223	2.3462	Cerebellum_Crus1_L
Positive	-24	-69	-24	223	2.3129	Cerebellum_6_L
Positive	-18	-81	-21	223	2.3087	Cerebellum_Crus1_L
Positive	48	15	33	57	2.3005	Frontal_Inf_Oper_R
Positive	-12	-87	-15	223	2.2299	Lingual_L
Positive	18	-75	-24	223	2.1739	Cerebellum_Crus1_R
Negative	54	0	6	236	-3.5945	Rolandic_Oper_R
Negative	48	-15	18	236	-3.3116	Rolandic_Oper_R
Negative	6	-72	9	188	-3.1327	Calcarine_R
Negative	36	6	12	236	-3.0721	Insula_R
Negative	45	-33	21	236	-2.9662	Temporal_Sup_R
Negative	-6	-21	48	387	-2.8871	Cingulum_Mid_L
Negative	-3	-75	6	188	-2.8325	Lingual_L
Negative	6	-3	45	387	-2.8206	Cingulum_Mid_R
Negative	6	6	39	387	-2.7774	Cingulum_Mid_R
Negative	-6	-3	45	387	-2.7639	Cingulum_Mid_L
Negative	3	-18	48	387	-2.7506	Cingulum_Mid_R
Negative	15	-75	-6	188	-2.6986	Lingual_R
Negative	-6	45	18	181	-2.6594	Cingulum_Ant_L
Negative	3	48	9	181	-2.6509	Cingulum_Ant_R
Negative	21	-84	-9	188	-2.6347	Lingual_R
Negative	0	-15	39	387	-2.6060	Cingulum_Mid_L
Negative	3	39	27	181	-2.5622	Cingulum_Ant_R
Negative	54	-27	21	236	-2.5489	Rolandic_Oper_R
Negative	-3	-15	66	387	-2.5459	Paracentral_Lobule_L
Negative	9	-69	0	188	-2.4945	Lingual_R
Negative	6	33	21	181	-2.4502	Cingulum_Ant_R
Negative	-6	57	9	181	-2.3199	Frontal_Sup_Medial_L
Negative	-12	-69	-3	188	-2.2153	Lingual_L
Negative	9	-30	51	387	-2.1977	Paracentral_Lobule_R
Negative	-3	27	27	181	-2.0930	Cingulum_Ant_L
Negative	-12	63	18	181	-2.0475	Frontal_Sup_Medial_L

CS: Cluster Size; AAL: Automated Anatomical Labeling

Table S3. Loadings for processing speed activation pattern scores.

Loading Direction	X	Y	Z	CS	bootstrap-Z	AAL_label
Positive	-21	-36	3	393	3.2376	Hippocampus_L
Positive	6	-12	33	112	2.8708	Cingulum_Mid_R
Positive	-27	-27	-27	393	2.8553	Fusiform_L
Positive	6	-21	33	112	2.7923	Cingulum_Mid_R
Positive	9	27	24	112	2.7541	Cingulum_Ant_R
Positive	-39	-33	-24	393	2.7407	Fusiform_L
Positive	0	9	27	112	2.6227	Cingulum_Ant_L
Positive	39	12	-24	82	2.5303	Temporal_Pole_Sup_R
Positive	-12	-27	6	393	2.4841	Thalamus_L
Positive	48	3	-12	82	2.4552	Temporal_Sup_R
Positive	-21	-18	-18	393	2.4428	ParaHippocampal_L
Positive	-33	-27	-15	393	2.4416	Fusiform_L
Positive	-27	-6	-6	393	2.4011	Putamen_L
Positive	-39	6	-9	393	2.3619	Insula_L
Positive	-6	-27	33	112	2.3268	Cingulum_Mid_L
Positive	-30	9	-6	393	2.2912	Putamen_L
Positive	-30	-3	-27	393	2.1814	Hippocampus_L
Positive	-48	-3	-6	393	2.1363	Temporal_Sup_L
Negative	0	-18	57	428	-3.2081	Supp_Motor_Area_L
Negative	9	-54	66	142	-2.9886	Precuneus_R
Negative	66	-24	18	170	-2.9560	Temporal_Sup_R
Negative	3	-9	51	428	-2.9085	Cingulum_Mid_R
Negative	0	-24	72	428	-2.7055	Paracentral_Lobule_R
Negative	3	3	54	428	-2.6914	Supp_Motor_Area_R
Negative	-3	-9	72	428	-2.6739	Supp_Motor_Area_L
Negative	-6	-63	63	142	-2.6710	Precuneus_L
Negative	-3	0	45	428	-2.6538	Cingulum_Mid_L
Negative	54	-15	51	80	-2.6374	Postcentral_R
Negative	0	0	69	428	-2.6087	Supp_Motor_Area_L
Negative	57	-24	48	80	-2.5683	SupraMarginal_R
Negative	-9	-51	69	142	-2.5105	Precuneus_L
Negative	57	-3	3	170	-2.4752	Temporal_Sup_R
Negative	42	-12	60	80	-2.3708	Precentral_R
Negative	39	-18	48	80	-2.3363	Postcentral_R
Negative	48	-33	24	170	-2.2871	Temporal_Sup_R
Negative	48	-6	54	80	-2.2355	Frontal_Mid_R
Negative	6	-63	63	142	-2.1867	Precuneus_R

CS: Cluster Size; AAL: Automated Anatomical Labeling

Table S4. Loadings for language activation pattern scores.

Loading Direction	X	Y	Z	CS	bootstrap-Z	AAL_label
Positive	6	-54	9	1701	3.7028	Lingual_R
Positive	9	-54	-3	1701	3.5977	Cerebellum_4_5_R
Positive	0	-63	39	1701	3.5483	Precuneus_L
Positive	3	-48	36	1701	3.4912	Cingulum_Mid_R
Positive	18	-54	-6	1701	3.3108	Lingual_R
Positive	3	-27	51	1701	3.3093	Cingulum_Mid_R
Positive	-9	-63	21	1701	3.2660	Calcarine_L
Positive	-3	-66	27	1701	3.2066	Cuneus_L
Positive	-3	48	-3	227	3.0747	Cingulum_Ant_L
Positive	-63	-30	15	162	3.0427	Temporal_Sup_L
Positive	0	-21	66	1701	3.0403	Supp_Motor_Area_R
Positive	3	-60	21	1701	3.0307	Cuneus_R
Positive	9	-51	69	1701	3.0204	Precuneus_R
Positive	-18	-51	-12	1701	3.0190	Cerebellum_4_5_L
Positive	66	-18	-3	211	2.9972	Temporal_Sup_R
Positive	0	-66	-21	1701	2.9872	Vermis_6
Positive	-15	-72	-18	1701	2.9776	Cerebellum_6_L
Positive	0	-51	57	1701	2.8753	Precuneus_L
Positive	-24	-75	-21	1701	2.8602	Cerebellum_6_L
Positive	63	-9	6	211	2.8256	Temporal_Sup_R
Positive	-63	-21	-3	162	2.7690	Temporal_Mid_L
Positive	-3	-66	60	1701	2.7580	Precuneus_L
Positive	-24	-60	-18	1701	2.7389	Cerebellum_6_L
Positive	9	-81	-15	63	2.7101	Cerebellum_6_R
Positive	12	-60	24	1701	2.6578	Precuneus_R
Positive	63	-30	15	211	2.6570	Temporal_Sup_R
Positive	0	-93	-9	63	2.6051	Calcarine_L
Positive	-6	-51	69	1701	2.5937	Precuneus_L
Positive	15	-30	0	1701	2.5814	Thalamus_R
Positive	54	-66	18	68	2.5560	Temporal_Mid_R
Positive	-9	-42	51	1701	2.5469	Cingulum_Mid_L
Positive	0	-39	69	1701	2.4991	Paracentral_Lobule_R
Positive	0	0	45	1701	2.4940	Cingulum_Mid_L
Positive	9	48	-6	227	2.4846	Frontal_Med_Orb_R
Positive	-63	-12	9	162	2.4234	Temporal_Sup_L
Positive	-57	-6	3	162	2.3086	Temporal_Sup_L
Positive	21	-42	-9	1701	2.3008	Lingual_R
Positive	57	-63	9	68	2.2327	Temporal_Mid_R
Positive	3	-60	63	1701	2.1421	Precuneus_R
Negative	9	24	39	2053	-4.0091	Cingulum_Mid_R
Negative	36	15	12	244	-3.7921	Insula_R
Negative	-51	0	33	2053	-3.5708	Precentral_L
Negative	12	27	30	2053	-3.5400	Cingulum_Ant_R
Negative	-9	3	66	2053	-3.5101	Supp_Motor_Area_L
Negative	30	24	6	244	-3.4966	Insula_R

Negative	9	24	51	2053	-3.4656	Supp_Motor_Area_R
Negative	-9	24	39	2053	-3.3652	Cingulum_Mid_L
Negative	-33	-48	45	248	-3.3632	Parietal_Inf_L
Negative	-39	0	45	2053	-3.3375	Precentral_L
Negative	-48	-57	-3	78	-3.3217	Temporal_Mid_L
Negative	3	6	33	2053	-3.3048	Cingulum_Mid_R
Negative	-3	12	27	2053	-3.2976	Cingulum_Ant_L
Negative	-24	3	51	2053	-3.2830	Frontal_Mid_L
Negative	45	-3	45	2053	-3.2622	Precentral_R
Negative	-6	12	57	2053	-3.2604	Supp_Motor_Area_L
Negative	-21	6	63	2053	-3.2324	Frontal_Sup_L
Negative	39	6	30	2053	-3.1890	Precentral_R
Negative	33	-45	42	191	-3.1754	Parietal_Inf_R
Negative	-6	36	51	2053	-3.1753	Frontal_Sup_Medial_L
Negative	-21	3	6	327	-3.1749	Putamen_L
Negative	-9	18	30	2053	-3.1722	Cingulum_Ant_L
Negative	27	48	18	2053	-3.1482	Frontal_Mid_R
Negative	9	15	60	2053	-3.1471	Supp_Motor_Area_R
Negative	-15	12	60	2053	-3.0941	Frontal_Sup_L
Negative	-15	9	9	327	-3.0571	Caudate_L
Negative	-27	-63	45	248	-3.0298	Parietal_Sup_L
Negative	27	-66	39	191	-3.0111	Occipital_Sup_R
Negative	45	27	27	2053	-2.9887	Frontal_Inf_Tri_R
Negative	-42	21	27	2053	-2.9649	Frontal_Inf_Tri_L
Negative	-21	12	51	2053	-2.9256	Frontal_Mid_L
Negative	12	3	63	2053	-2.8347	Supp_Motor_Area_R
Negative	-57	-45	-15	78	-2.8127	Temporal_Inf_L
Negative	-42	27	18	2053	-2.7977	Frontal_Inf_Tri_L
Negative	-51	21	30	2053	-2.7973	Frontal_Inf_Tri_L
Negative	51	21	33	2053	-2.7830	Frontal_Inf_Oper_R
Negative	-9	27	24	2053	-2.6879	Cingulum_Ant_L
Negative	12	36	18	2053	-2.6857	Cingulum_Ant_R
Negative	-12	-15	3	60	-2.6235	Thalamus_L
Negative	42	12	-12	244	-2.6090	Insula_R
Negative	54	0	30	2053	-2.6085	Postcentral_R
Negative	-27	-12	0	327	-2.5915	Pallidum_L
Negative	6	-15	33	2053	-2.5736	Cingulum_Mid_R
Negative	9	-27	36	2053	-2.5710	Cingulum_Mid_R
Negative	-18	-66	57	248	-2.5440	Parietal_Sup_L
Negative	-39	-33	-24	78	-2.5050	Fusiform_L
Negative	9	-36	33	2053	-2.4009	Cingulum_Mid_R
Negative	-54	-45	-3	78	-2.3787	Temporal_Mid_L
Negative	39	36	21	2053	-2.3445	Frontal_Mid_R
Negative	-18	-24	12	60	-2.3153	Thalamus_L
Negative	63	3	27	2053	-2.3052	Precentral_R
Negative	-48	6	18	2053	-2.3016	Precentral_L
Negative	24	0	3	244	-2.2909	Pallidum_R

Negative	48	12	18	2053	-2.2772	Frontal_Inf_Oper_R
Negative	36	3	57	2053	-2.2706	Frontal_Mid_R
Negative	18	-72	45	191	-2.2257	Precuneus_R
Negative	-9	39	30	2053	-2.2115	Frontal_Sup_Medial_L
Negative	-30	21	45	2053	-2.1859	Frontal_Mid_L
Negative	33	15	48	2053	-2.1383	Frontal_Mid_R

CS: Cluster Size; AAL: Automated Anatomical Labeling

Supplemental References

1. Habeck C, Eich T, Razlighi R, Gazes Y, Stern Y. Reference ability neural networks and behavioral performance across the adult life span. Neuroimage. 2018;172:51-63.
2. Habeck C, Gazes Y, Razlighi Q, Steffener J, Brickman A, Barulli D, et al. The Reference Ability Neural Network Study: Life-time stability of reference-ability neural networks derived from task maps of young adults. Neuroimage. 2016;125:693-704.
3. Habeck C, Stern Y, Alzheimer's Disease Neuroimaging I. Multivariate data analysis for neuroimaging data: overview and application to Alzheimer's disease. Cell Biochem Biophys. 2010;58(2):53-67.
4. Habeck C, Rakitin BC, Moeller J, Scarmeas N, Zarahn E, Brown T, et al. An event-related fMRI study of the neurobehavioral impact of sleep deprivation on performance of a delayed-match-to-sample task. Brain Res Cogn Brain Res. 2004;18(3):306-21.
5. Habeck C, Hilton HJ, Zarahn E, Flynn J, Moeller J, Stern Y. Relation of cognitive reserve and task performance to expression of regional covariance networks in an event-related fMRI study of nonverbal memory. Neuroimage. 2003;20(3):1723-33.
6. Moeller JR, Strother SC, Sidtis JJ, Rottenberg DA. Scaled subprofile model: a statistical approach to the analysis of functional patterns in positron emission tomographic data. J Cereb Blood Flow Metab. 1987;7(5):649-58.
7. Burnham KP, Anderson DR, Burnham KP. Model selection and multimodel inference : a practical information-theoretic approach. 2nd ed. New York: Springer; 2002. xxvi, 488 p. p.
8. Burnham KP, Anderson DR. Model selection and inference : a practical information-theoretic approach. New York: Springer; 1998. xix, 353 p. p.
