# Supplementary material for: Domain-specific associations of metabolomic features with cognition and neural networks among cognitively normal adults
Source: Front Neurosci. 2026 Oct 20;20:1898605. doi: 10.3389/fnins.2026.1898605 (PMC13727718; doi:10.3389/fnins.2026.1898605)

Supplemental Results

Figure S2. Scree plot showing the proportion of total variance explained by each principal component from a principal component analysis of 4,286 metabolites measured in 112 participants.

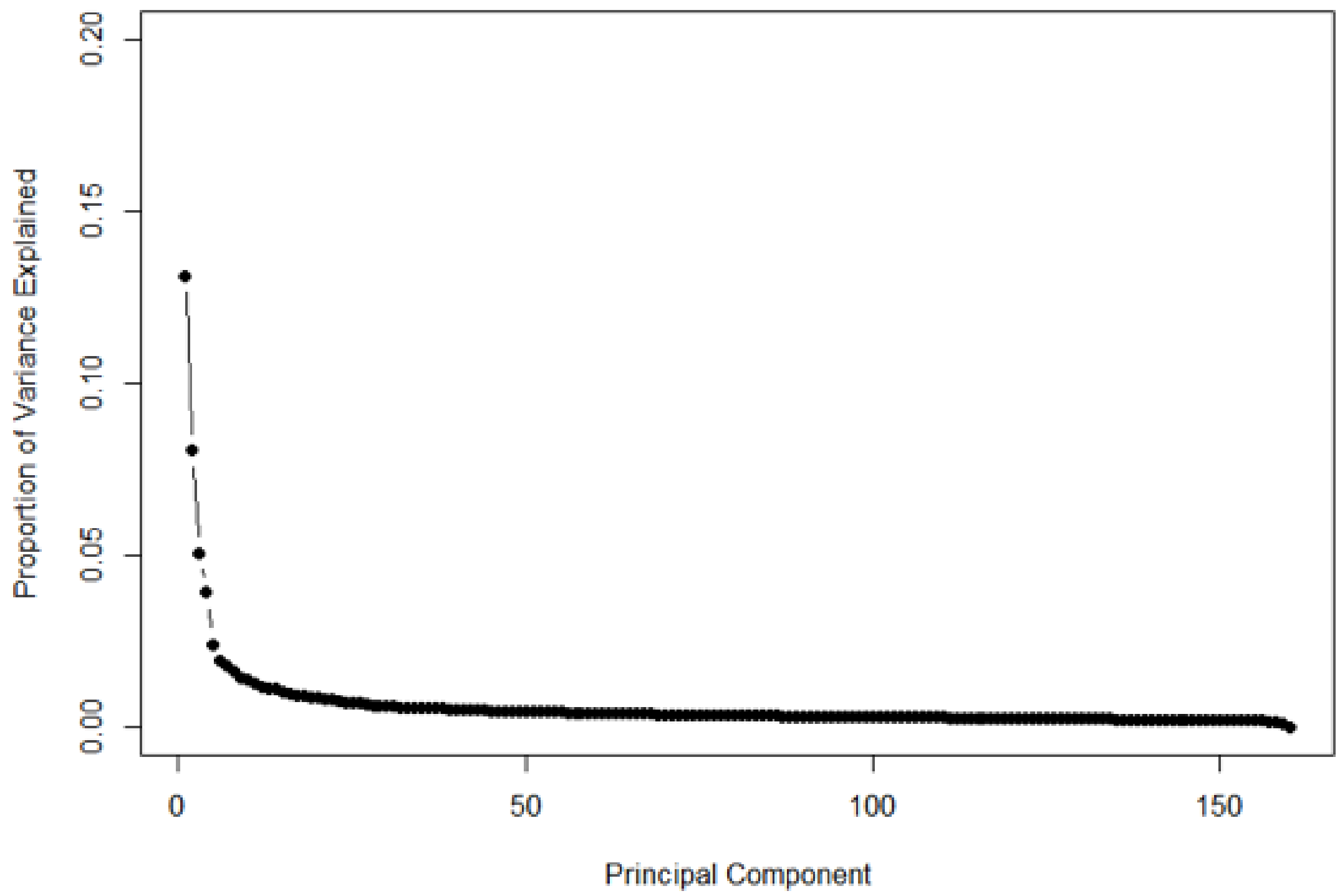

Table S5. Sensitivity Analysis of Effect Estimate Robustness to Metabolite Values Below the Limit of Detection

Cognition	
**Domain**	**Correlation**
Memory	0.800
Reason	0.831
Speed	0.696
Vocab	0.779
**Neural Networks**	
**Domain**	**Correlation**
Memory	0.814
Reason	0.764
Speed	0.663
Vocab	0.840

Table S6. Metabolite features associated with all cognitive domains, or all domain-specific network activation pattern scores in our exploratory analysis.

**Cognition**					
**M/Z**	**Time (s)**	**Annotation**	**Formula**	**Confidence Level**	**ESI**
193.0171	99.8	Sequoyitol<br>Mytilitol<br>Methyl beta-D-glucopyranoside<br>L-Quebrachitol<br>L-Pinitol<br>D-Pinitol<br>D-4-O-Methyl-myo-inositol<br>4-O-Methyl-myo-inositol<br>(-)-Bornesitol	C7H14O6	Level 3	-
**Neural Network Activation**					
**M/Z**	**Time (s)**	**Annotation**	**Formula**	**Confidence Level**	**ESI**
198.098	30.6	Isepamicin	C22H43N5O12	Level 5	+
528.7699	57.4	Unknown	Unknown		+
199.0947	29.0	Fluticasone 17beta-carboxylic acid	C21H26F2O5	Level 5	+
199.1012	29.9	Ganoderic acid H	C32H44O9	Level 5	+
385.3459	23.4	7-Dehydrocholesterol<br>Desmosterol	C27H44O	Level 1	+
766.5745	26.2	PC(o-16:0/20:5)<br>PC(o-16:1/20:4)<br>PC(P-18:1/18:3)<br>PC(P-18:4/18:0)<br>PC(P-16:0/20:4)	C44H80NO7P	Level 3	+

Table S7. Pathway analysis for the domain of memory.

Cognition						
**Pathway**	**Pathway Total**	**Significant Hits**	**Expected**	**Enrichment Ratio**	**p-value**	**FDR Q**
Valine, leucine and isoleucine degradation	65	20	7.0393	1.704715	0.001152	0.093336
Histidine metabolism	33	17	3.5738	2.798142	0.003742	0.299384
Butanoate metabolism	34	20	3.6821	2.987426	0.004788	0.378244
Beta-Alanine metabolism	20	11	2.1659	3.231913	0.008807	0.686977
Nitrogen metabolism	6	5	0.64978	6.15593	0.017366	1
Lysine metabolism	52	21	5.6315	1.775726	0.024517	1
Urea cycle/amino group metabolism	85	39	9.2053	1.738129	0.025136	1
**Neural Network Activation**						
**Pathway**	**Pathway Total**	**Significant Hits**	**Expected**	**Enrichment Ratio**	**p-value**	**FDR Q**
Arginine and Proline Metabolism	45	19	4.4385	4.280725	3.69E-05	0.002691
Valine, leucine and isoleucine degradation	65	11	6.4112	1.715747	0.002142	0.154224
Aspartate and asparagine metabolism	114	23	11.244	2.045535	0.003632	0.257858
Butanoate metabolism	34	10	3.3535	2.981959	0.008331	0.58317
Urea cycle/amino group metabolism	85	15	8.3839	1.789143	0.02429	1
Beta-Alanine metabolism	20	6	1.9727	3.041517	0.025034	1
Propanoate metabolism	31	7	3.0576	2.289377	0.026997	1
Glycine, serine, alanine and threonine metabolism	88	16	8.6798	1.84336	0.034998	1
Lysine metabolism	52	9	5.129	1.754728	0.037814	1
Lipoate metabolism	8	3	0.78907	3.801944	0.042461	1

Enrichment Factor : (Observed significant hits in a pathway) / (Expected hits by chance)
FDR: False discovery rate

Figure S3. Mummichog Pathway Activity Profile for memory domain.

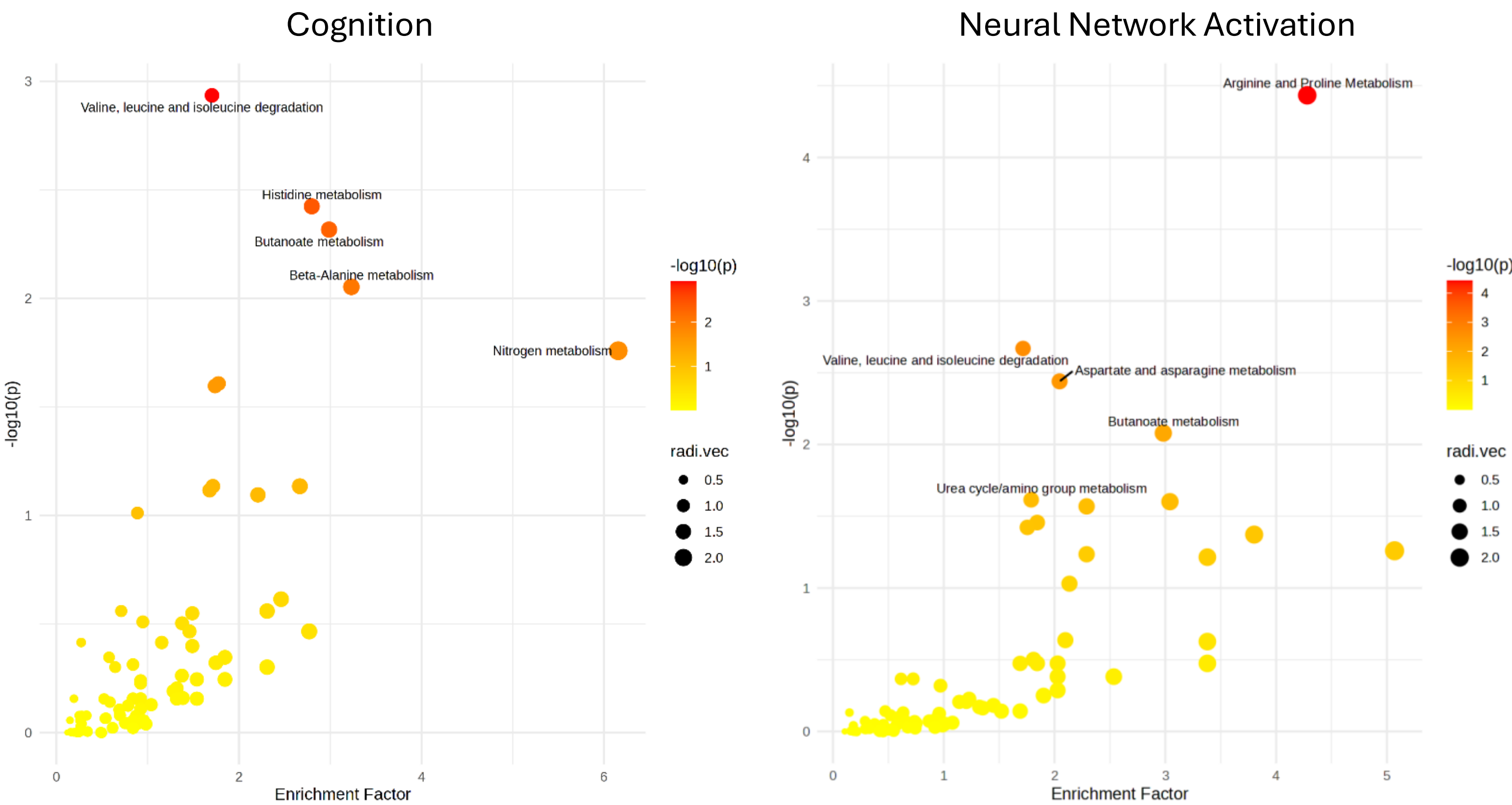

Table S8. Pathway analysis for the domain of reasoning.

Cognition						
**Pathway**	**Pathway Total**	**Significant Hits**	**Expected**	**Enrichment Ratio**	**p-value**	**FDR Q**
Arginine and Proline Metabolism	45	12	3.9137	3.066152	0.033281	1

Neural Network Activation						
**Pathway**	**Pathway Total**	**Significant Hits**	**Expected**	**Enrichment Ratio**	**p-value**	**FDR Q**
Fatty acid activation	74	14	3.9207	3.570791	3.20E-05	0.00208
De novo fatty acid biosynthesis	106	11	5.6161	1.958655	0.000131	0.008372
Fatty acid oxidation	35	7	1.8544	3.774806	0.000205	0.012921
Fatty acid oxidation, peroxisome	28	4	1.4835	2.696326	0.00299	0.185374
Di-unsaturated fatty acid beta-oxidation	26	3	1.3775	2.177858	0.00717	0.437358
Omega-3 fatty acid metabolism	39	4	2.0663	1.935827	0.039472	1
Mono-unsaturated fatty acid beta-oxidation	19	2	1.0067	1.986689	0.043557	1
Polyunsaturated fatty acid biosynthesis	21	2	1.1126	1.797591	0.043557	1

Enrichment Factor : (Observed significant hits in a pathway) / (Expected hits by chance)
FDR: False discovery rate

Figure S4. Mummichog Pathway Activity Profile for reasoning domain.

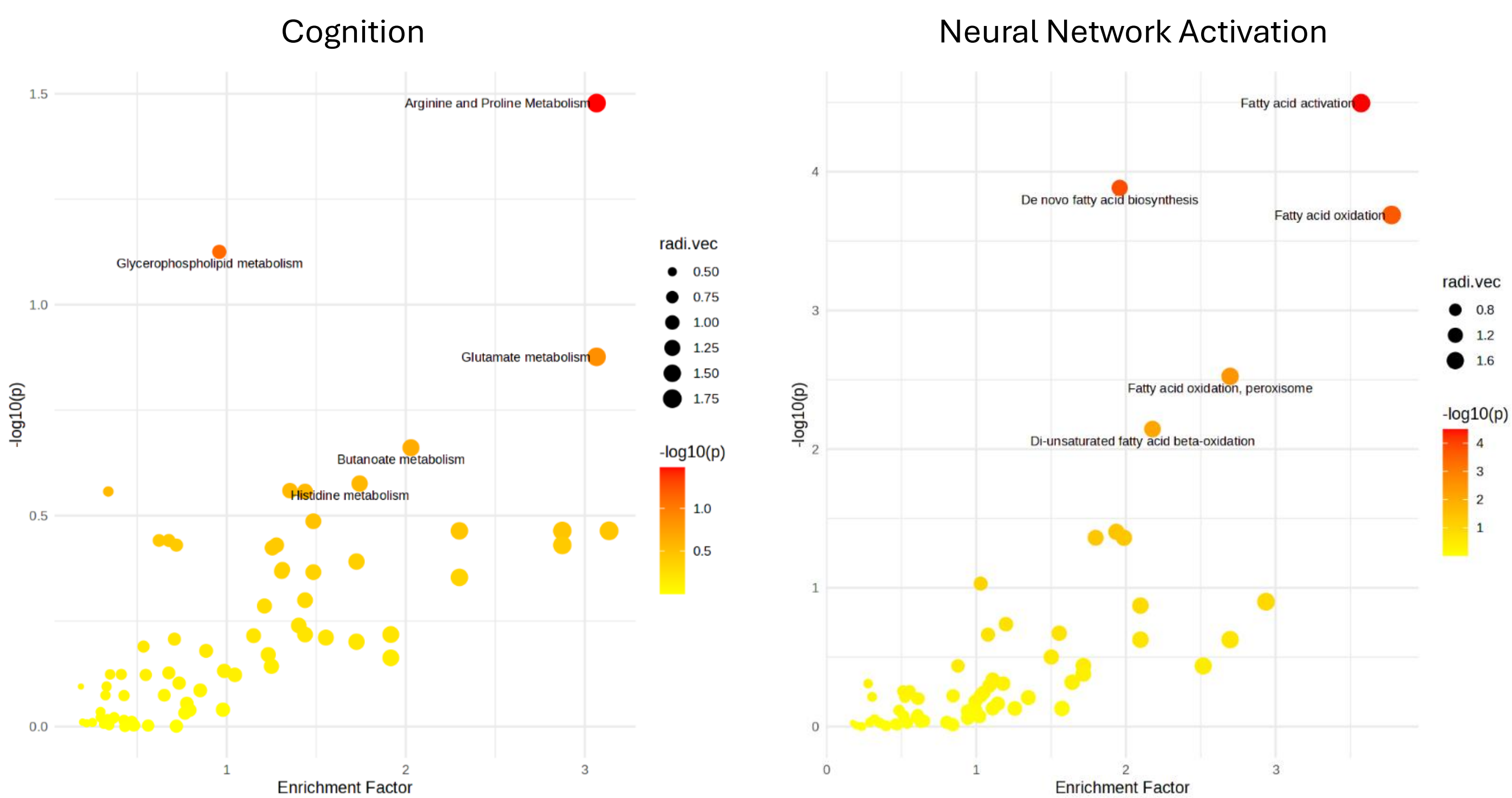

Table S9. Pathway analysis for the domain of speed.

Pathway	Pathway Total	Significant Hits	Expected	Enrichment Ratio	p-value	FDR Q
**Cognition**						
Alanine and Aspartate Metabolism	30	8	1.6195	4.939796	0.001371	0.08223
Arginine and Proline Metabolism	45	11	2.4292	4.52824	0.002284	0.134744
Pyruvate Metabolism	20	6	1.0796	5.557614	0.007646	0.443439
Nitrogen metabolism	6	3	0.32389	9.262404	0.017181	0.979317
Ascorbate (Vitamin C) and Aldarate Metabolism	29	6	1.5655	3.832641	0.020366	1
Urea cycle/amino group metabolism	85	10	4.5885	2.179361	0.02117	1
Glyoxylate and Dicarboxylate Metabolism	12	4	0.64778	6.174936	0.029681	1
**Neural Network Activation**						
**Pathway**	**Pathway Total**	**Significant Hits**	**Expected**	**Enrichment Ratio**	**p-value**	**FDR Q**
Biopterin metabolism	22	5	1.9793	2.526146	0.002169	0.167044
Alanine and Aspartate Metabolism	30	10	2.6991	3.704939	0.002533	0.192508
Selenoamino acid metabolism	35	4	3.149	1.270245	0.041752	1
Valine, leucine and isoleucine degradation	65	8	5.8481	1.367966	0.045352	1
Vitamin H (biotin) metabolism	5	2	0.44985	4.445926	0.045785	1

Enrichment Factor : (Observed significant hits in a pathway) / (Expected hits by chance)
FDR: False discovery rate

Figure S5. Mummichog Pathway Activity Profile for speed domain.

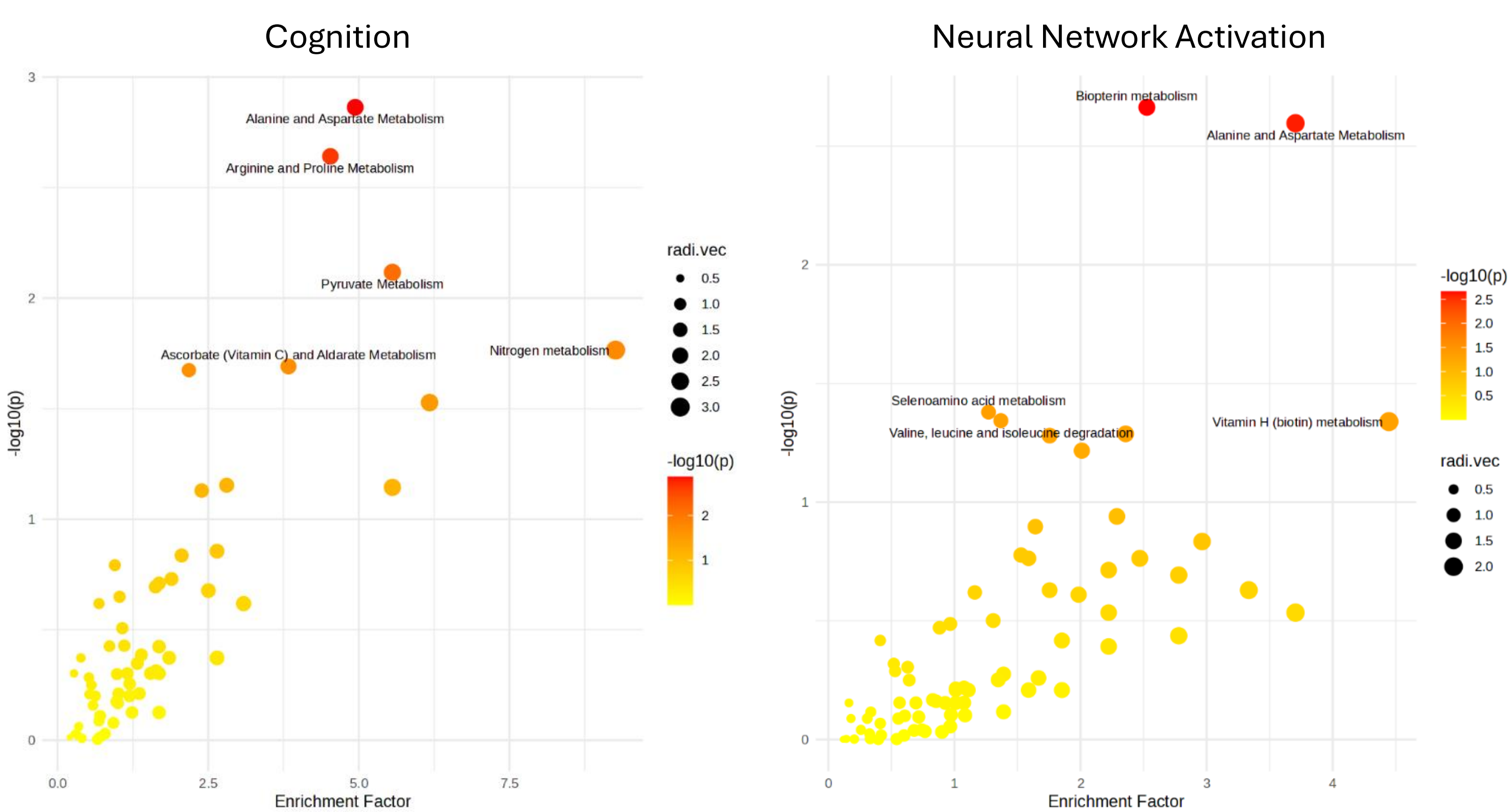

Table S10. Pathway analysis for the domain of language.

Pathway	Pathway Total	Significant Hits	Expected	Enrichment Ratio	p-value	FDR Q
**Cognition**						
**Pathway**	**Pathway Total**	**Significant Hits**	**Expected**	**Enrichment Ratio**	**p-value**	**FDR Q**
Histidine metabolism	33	9	4.2556	2.11486	0.045387	1
**Neural Network Activation**						
**Pathway**	**Pathway Total**	**Significant Hits**	**Expected**	**Enrichment Ratio**	**p-value**	**FDR Q**
Pyrimidine metabolism	70	13	6.1813	2.103117	0.015194	1

Enrichment Factor : (Observed significant hits in a pathway) / (Expected hits by chance)
FDR: False discovery rate

Figure S6. Mummichog Pathway Activity Profile for language domain.

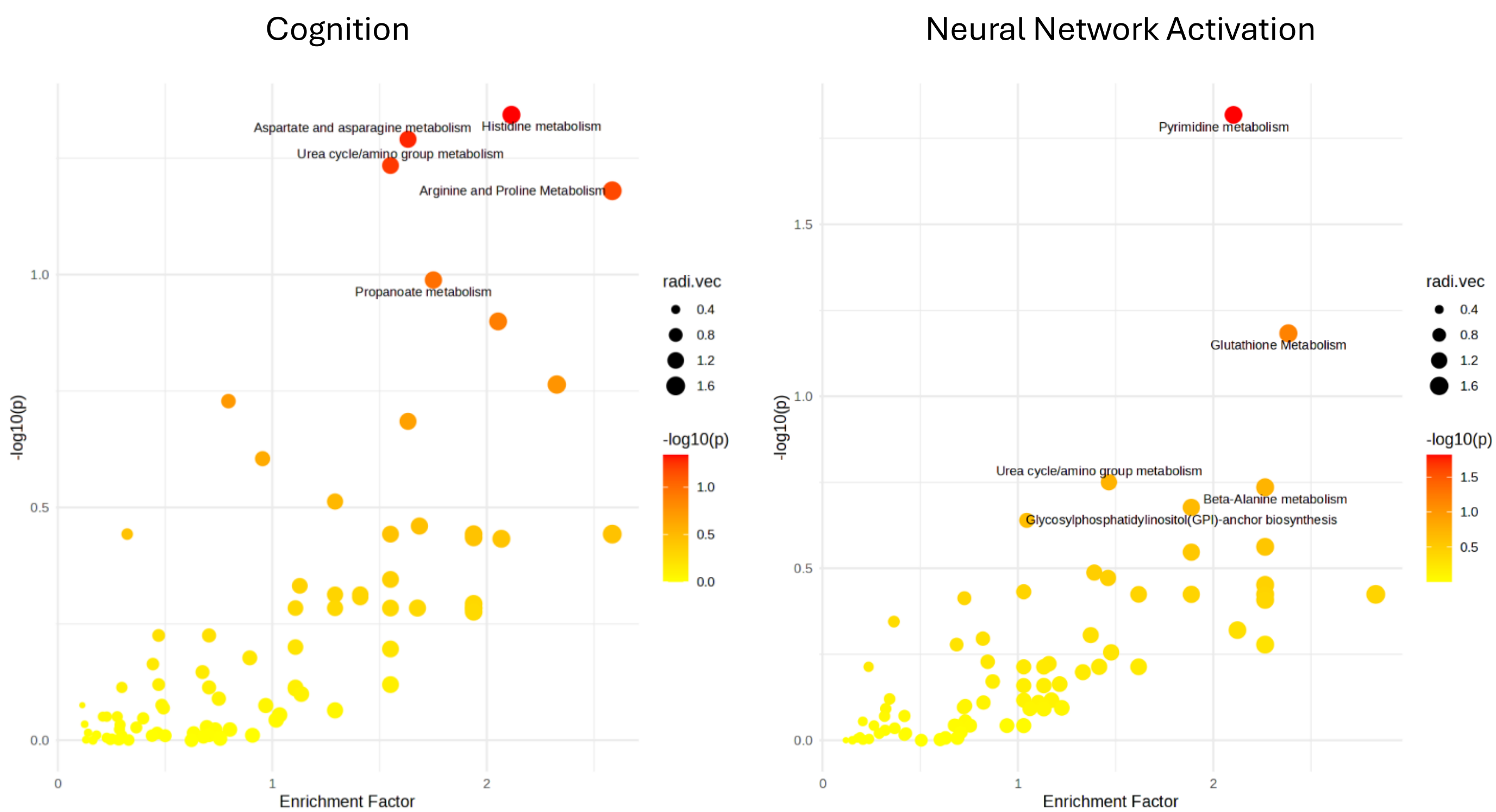

Supplement: Supplementary file 2 [file Data_Sheet_2.pdf]
